# Exercise, Mediterranean Diet Adherence or Both during Pregnancy to Prevent Postpartum Depression—GESTAFIT Trial Secondary Analyses

**DOI:** 10.3390/ijerph192114450

**Published:** 2022-11-04

**Authors:** Marta Flor-Alemany, Jairo H. Migueles, Inmaculada Alemany-Arrebola, Virginia A. Aparicio, Laura Baena-García

**Affiliations:** 1 Department of Physiology, University of Granada, 18071 Granada, Spain; 2 Institute of Nutrition and Food Technology (INYTA), Biomedical Research Centre (CIBM), University of Granada, 18016 Granada, Spain; 3 Sport and Health University Research Institute (IMUDS), 18007 Granada, Spain; 4 Department of Biosciences and Nutrition, Karolinska Institutet, 141 52 Huddinge, Sweden; 5 PROFITH “Promoting FITness and Health through Physical Activity” Research Group, Department of Physical Education and Sports, Faculty of Sport Sciences, University of Granada, 18071 Granada, Spain; 6 Department of Developmental and Educational Psychology, Faculty of Education and Sports Sciences, University of Granada, 52005 Melilla, Spain; 7 Department of Nursing, Faculty of Health Sciences, University of Granada, 51001 Ceuta, Spain

**Keywords:** depression, diet, Mediterranean, exercise, postpartum period, pregnant women

## Abstract

Targeting lifestyle behaviors during pregnancy is crucial to prevent the highly prevalent postpartum depression and its consequences. In these secondary analyses of an intervention trial to investigate the effects of concurrent exercise training on postpartum depression, we aimed to investigate the potential role of Mediterranean diet (MD) adherence on the exercise effects. A total of 85 pregnant women met the per-protocol criteria (exercise n = 46, control n = 39). The exercise program was delivered in 60 min sessions, 3 days/week, from the 17th gestational week until birth. Women’s dietary habits were assessed with a food frequency questionnaire. The Mediterranean Food Pattern (an MD index) was derived from it to assess MD adherence. We used the Edinburgh Postnatal Depression Scale to assess postpartum depression. The postpartum depression score was not statistically different between control and exercise groups (*p* > 0.05). A higher consumption of fruits (β = −0.242, *p* = 0.022), lower intake of red meat and subproducts (β = 0.244, *p* = 0.020), and a greater MD adherence (β = −0.236, *p* = 0.027) were associated with lower levels of postpartum depression. Greater adherence to the MD during pregnancy was associated with fewer depressive symptoms and a lower risk of postpartum depression. Postnatal depression was not reduced by prenatal exercise. Promoting fruit consumption while controlling the intake of red meat during pregnancy might prevent postnatal depression.

## 1. Introduction

Globally, between 10 and 15% of women are affected by depression during the postpartum period [1]. Postpartum depression typically onsets between the 4th–6th week after giving birth, and it is identified as a critical public health problem [2,3]. In fact, postnatal depression might chronify after giving birth, potentially harming the mother and children later in life [2,3]. For instance, it might increase the risk for sadness, anxiety, and failure to initiate and/or maintain breastfeeding in the mother [4], which may affect the mother-to-child relationship [5]. Furthermore, children of mothers with depression are at a higher risk of being underweight and having stunted growth in the first year [6], and of suffering from an array of physical and psychological conditions [5,7]. 

Interventions before childbirth have a huge importance in the prevention of the highly prevalent postpartum depression and its potential consequences. In this context, exercise and healthy diets might partially offset depressive symptoms in the non-pregnant adult population [8,9,10]. Exercise during pregnancy seems to lower the prevalence of postpartum depression [11] and depressive symptoms [12]. A recent systematic review of meta-analyses established that exercise had a significant, small effect on postpartum depressive symptoms [13], although this is inconclusive, since previous exercise programs of both aerobic and strengthening exercises did not find a protective effect on postpartum depression [14,15,16]. Regarding diet, the Mediterranean diet (MD) is the dietary pattern with more supporting evidence for its effects on depression in adults [10]. However, the extrapolation of these benefits to pregnant women is not straightforward and previous literature reports mixed findings [17,18].

Thus, it is warranted to study the combined effect of a healthy nutritional pattern (i.e., MD adherence) together with exercise adapted to pregnancy throughout gestation, to explore the potential positive moderating role of MD adherence on the exercise effects. Therefore, the aim of these secondary analyses from the GEStation and FITness (GESTAFIT) project [19,20] was threefold: (1) to explore the effects of an exercise intervention delivered during pregnancy on postpartum depression; (2) to investigate the association of MD adherence during pregnancy with postpartum depression; and (3) to check whether following a Mediterranean diet during pregnancy moderates the effects of exercise on postpartum depression.

## 2. Materials and Methods

### 2.1. Study Design and Participants

This study includes secondary analyses from GESTAFIT, an exercise-based trial on pregnant women. The GESTAFIT protocol has been previously published (Identifier: NCT02582567) [19]. The study was conducted at the “Sport and Health University Research Institute” (Granada, Spain) and at the “San Cecilio and Virgen de las Nieves University Hospitals” from November 2015 to April 2018. Of 384 pregnant women assessed for eligibility, 159 women met the inclusion–exclusion criteria (Supplementary Table S1). Among them, a total of 85 women who had valid data in sociodemographic characteristics, MD adherence at the 16th gestational week (g.w.), and postpartum depression assessed at the 6th week after giving birth were included for this analysis (Supplementary Figure S1). All participants provided signed informed consent, and the study was approved by the Clinical Research Ethics Committee of Granada, Government of Andalusia, Spain (code: GESFIT-0448-N-15).

### 2.2. Sample Size Calculation

A priori sample size calculations were only performed for the primary outcome of GESTAFIT (i.e., gestational weight gain), which resulted in 52 pregnant women (26 per study arm). Notwithstanding, a posteriori sample size calculations considering the sample size of this study (n = 85) showed a statistical power of 80% to detect small-to-medium association sizes (f^2^ ≥ 0.12). Statistical power analyses were performed using G*Power 3.1 [21]. 

### 2.3. Randomization and Blinding

The GESTAFIT project was conducted in three waves for feasibility reasons. Upon an observed high dropout rate in the control group at the beginning of the study, the initial study design (randomized control trial) was partially broken in the second and third waves of participants, which has been frequently reported in research exercise during pregnancy [22,23]. As a result, half the women were not randomised but allocated according to their convenience. Neither the gynaecologists, midwives from hospitals, nor the research team who was responsible for the assessment were aware of participants’ group allocation. 

### 2.4. Exercise Group

The exercise training program has been extensively detailed elsewhere [19]. In brief, it followed the standards of the American College of Obstetricians and Gynecologists [24] and the latest scientific evidence in this field of research [25]. From the 17th g.w. until birth, the exercise program consisted of both aerobic and strength exercises (60 min session, three days per week). The exercises were of moderate intensity, according to the rating of the perceived effort scale reported by the participants (i.e., 12–16 points out of a range from 6 to 20, using the Borg scale) [26]. To determine compliance to the exercise program, the participants’ attendance at the exercise sessions was tracked.

### 2.5. Control Group

Pregnant women in the control group were asked to keep their regular activities and were not invited to the training sessions. Nevertheless, the research team organized a number of workshops on physical exercise and healthy eating practices during pregnancy for ethical considerations. These workshops were delivered to the control and exercise groups.

### 2.6. Postpartum Depression

Postpartum depression was determined with the Spanish version of the Edinburgh Postnatal Depression Scale (EPDS) [27,28]. This scale consists of 10 Likert-style items that range from 0 to 3, which results in a final score of 0 to 30 (higher scores reflect higher severity of depressive symptoms). An EPDS score of 10/11 out of 30 has been suggested to detect major and minor depression, with a sensitivity of 79%, and specificity of 95.5% (the EDPS Cronbach’s α was 0.87). The EPDS was considered a continuous indicator of depressive symptoms and, concomitantly, categorized to detect postpartum depression as an EPDS score ≥ 10.

### 2.7. Dietary Assessment and Mediterranean Diet Adherence

To assess dietary habits, we employed a food frequency questionnaire validated in Spanish adults from the Andalusian region [29]. The Mediterranean Food Pattern (a MD adherence index) was then calculated using the food frequency questionnaire, as it has previously been done in this study population [30]. The Mediterranean Food Pattern [31] includes eight components: olive oil, fiber, fruits, vegetables, fish, cereals, meat, and alcohol, which are quantified on a Likert scale ranging from 1 to 5. This results in a total score of 5–40, yet alcohol consumption was not considered, as it is contraindicated in pregnant women. As a result, the range of the total score in this study is 4 to 35, with higher values indicating greater adherence to MD.

### 2.8. Sociodemographic Characteristics

At the 16th g.w., participants’ sociodemographic and clinical characteristics (i.e., age, educational, marital, and working status, number of miscarriages, smoking habit, and diagnosis of depression/anxiety) were assessed with a self-reported questionnaire (anamnesis). 

### 2.9. Lower Back Pain

Lower back pain was assessed with the Pain Visual Analogue Scale [32] as the measured distance (mm) in a 100 mm line going from 0 (i.e., “no pain”) to the participants’ mark.

### 2.10. Physical Activity Levels

To objectively measure physical activity levels at the 16th and 34th g.w., accelerometry was employed. Women were asked to wear a tri-axial accelerometer attached to their non-dominant wrist (Actigraph GT3X+, Pensacola, FL, USA) for nine consecutive days. Total physical activity (min/week) was calculated.

### 2.11. Statistical Analyses

Participants’ characteristics at baseline were summarized as mean (standard deviation) or frequency (%), as appropriate. We used one-way analysis of covariance (ANCOVA) to explore the between-group differences in postpartum depression. As it was defined in the study protocol [19], the analyses were conducted under the per-protocol principles. 

Hierarchical linear regression was used to investigate the associations of dietary habits and MD adherence during gestation with postpartum depression. The stepwise method was used, introducing potential confounders in step 1 to test their association with postpartum depression (outcome). The choice of potential confounders was based on a previous study which was conducted in the same sample [33], where we explored the association of potential confounders with mental health outcomes (i.e., age, having a diagnosis of depression or anxiety, number of abortions, smoking habit, gestational weight gain, lower back pain, marital status, and educational level). Step 1 was performed to select the relevant confounders that explain a significant amount of variance in postpartum depression (see Table 3 footnotes). Next, dietary habits and MD adherence during pregnancy and postpartum were included in step 2 and were included in the models together with the confounders that were kept in step 1.

We explored the odds ratio of having postpartum depression (EPDS ≥ 10) as a function of the experimental group (exercise vs. control), MD adherence (below vs. above-median), and the potential moderating role of MD adherence on the exercise effects. The association between dietary habits and postpartum depression was assessed by logistic regression, obtaining the odds ratios (ORs). The Statistical Package for Social Sciences (IBM SPSS Statistics for Windows, version 22.0, Armonk, NY, USA) was used for the analyses, with the level of significance assumed to be at *p* < 0.05.

## 3. Results

Of the 159 pregnant women who met the eligibility criteria (Supplementary Table S1), 85 had available data on dietary habits, MD adherence, sociodemographic and clinical characteristics, and postpartum depression, and were included for the present analysis (Supplementary Figure S1). Table 1 shows the characteristics of the study participants. In brief, 42% of the pregnant women had one or more miscarriages in the past. In addition, more than half of the participants (69%) had a university degree or higher, were married or with a partner (61%), and were working (72%). The prevalence of postpartum depression based on the EDPS score ≥ 10 was 17%. No differences between exercise and control groups were found at baseline (all, *p* > 0.05). We did not observe any effect of the exercise intervention on the physical activity levels of the participants (*p* = 0.409). Women in both the control and the exercise group reduced their lifestyle physical activity during the course of pregnancy by 47 min/week on average (i.e., similar reduction in both groups).

Postpartum depression according to exercise intervention (control vs. exercise groups) is shown in Table 2. Although non-significant, in a per-protocol basis analysis, the exercise group showed a reduction in postpartum depression scores (EPDS score difference = −1.327 [95% CI: −3.099 to 0.446], *p* = 0.140).

Hierarchical linear regression analyses on dietary habits and MD adherence and postpartum depression are presented in Table 3. The intake of fruits was negatively associated (β = −0.242, *p* = 0.022), and the intake of red meat and subproducts was positively associated (β = 0.244, *p* = 0.020) with postpartum depression. Likewise, a greater MD adherence (β = −0.236, *p* = 0.027) was associated with lower postpartum depression.

**Figure 1:**
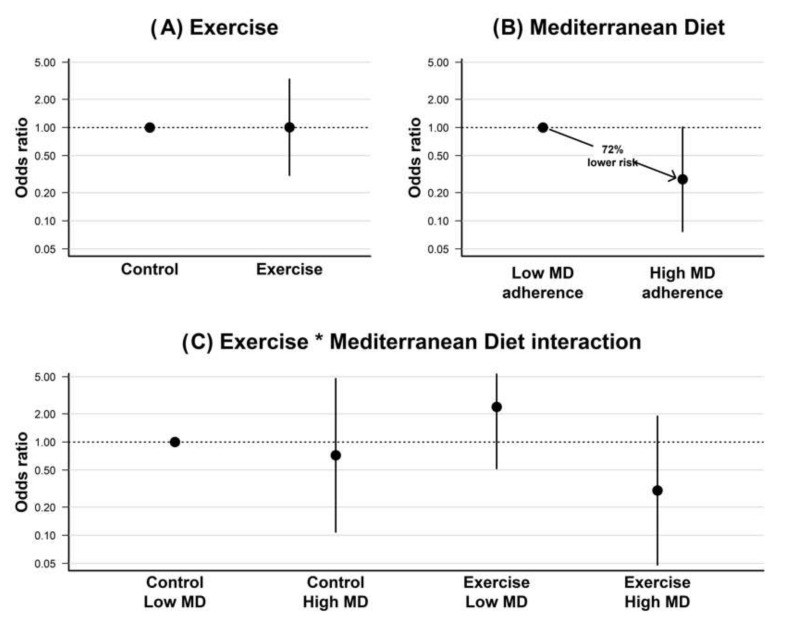
Odds ratio for postpartum depression relative to (**A**) experimental group (exercise vs. control), (**B**) Mediterranean diet adherence (above vs. below-median), and (**C**) the interaction between exercise and Mediterranean diet adherence. * represents the interaction term.

The odds ratio for presenting postpartum depression (EPDS ≥ 10) as a function of the exercise (exercise vs. control group), the MD adherence (below vs. above the median), and the the potential moderating role of MD adherence on the exercise effects are graphically shown in Figure 1. The exercise intervention had a null effect on postpartum depression (OR = 1.005, 95% CI: 0.310 to 3.294, *p* = 0.993). An optimal MD adherence during pregnancy (above-median) was associated with a 72% lower risk for postpartum depression (OR = 0.278, 95% CI: 0.077 to 1.009, *p* = 0.052). Regarding the potential moderating role of MD adherence on the exercise effects, those participants who received the exercise intervention and had a greater MD adherence (i.e., higher median split) had a lower non-significant risk for postpartum depression relative to participants in the control group and below the median MD adherence (OR = 0.302, 95% CI: 0.048 to 1.892, *p* = 0.201). 

## 4. Discussion

The findings of this two-arm study of counselling vs. exercising groups suggest that exercising during pregnancy did not significantly affect the risk for postpartum depression. However, a greater MD adherence, including a higher consumption of fruits and lower consumption of red meat and subproducts during pregnancy, was associated with fewer depressive symptoms and a lower likelihood of depression in the early postpartum.

The prevalence of postpartum depression in our sample was 17%, which matches the estimated rate in the Spanish population (i.e., 15% to 22%) [28,34]. Postpartum depression can affect maternal and child health [1]. Thus, establishing effective, safe, inexpensive, and well-accepted interventions to prevent and treat postpartum depression is paramount [9].

Exercise may prove to be an effective non-pharmacological way for women to regulate mood states following pregnancy [35]. Aerobic and strength training are effective in decreasing depression symptoms and enhancing the positive mood in people with a diagnosis of depression [8]. However, research examining exercise during pregnancy to prevent postpartum depression is inconclusive [36,37]. According to a systematic review, exercise reduced postpartum depression assessed with the EPDS by four points [36]. On the contrary, a more recent systematic review did not find evidence of the exercise potential to reduce postnatal depressive symptoms [37]. Meta-analytic data showed a small effect of exercise on postpartum depression [13]. In contrast, our findings show no effect of exercise on postpartum depression, which might be explained by the lack of statistical power, since our findings go in the same direction as that observed in the previous meta-analysis, yet findings in the GESTAFIT trial did not reach statistical significance.

Previous trials [11,14,16] have focused on the effects of an exercise intervention program during gestation on the risk of developing postpartum depression. Similar to our results, two previous trials [14,16] observed a non-statistically significant reduction in the EDPS score in the exercise group compared to controls (from 16–20th g.w. to 32–36th g.w.). Similarly, Vargas-Terrones et al. [11] observed a significant reduction in the risk for postpartum depression in the exercise group compared with controls. These discrepancies could be attributable to differences in the exercise program conducted and the tools employed to assess postpartum depression. First, we monitored the exercise intensity according to participants’ perceived exertion, which is considered valid during pregnancy [38]. However, common concerns regarding exercise during pregnancy might influence women’s perceived exertion [39]. Nonetheless, Vargas-Terrones et al. [11] monitored the intensity of the sessions with a heart rate monitor, targeting a 55–60% heart rate reserve during the sessions. Moreover, the prevalence of postpartum depression reported by Vargas-Terrones et al. [11] was 30% in the control group and 15% in the exercise group. Notwithstanding, the prevalence of postpartum depression in the studies conducted by Coll et al. [16] and Songøygard et al. [14] ranged from 4% to 9%, while we found a prevalence that ranged from 15% to 17%. It might be that exercise is particularly effective in those populations with higher rates of depressive symptoms. Vargas-Terrones et al. [11] employed the Center for Epidemiologic Studies Depression scale (CES-D), while Coll et al. [16] and Songøygard et al. [14] employed the same questionnaire that we employed in our study (i.e., EPDS). The CES-D has been showed to overestimate postpartum depression compared with the EPDS [40], which could explain why Vargas-Terrones et al. [11] reported a higher prevalence of postpartum depression. Furthermore, we observed a higher dropout rate in controls (55%) than in the exercise group (29%), which may have affected the between-group comparability of the prevalence of higher EPDS scores. This lower prevalence might have resulted in lower statistical power to detect differences. Therefore, the present findings must be interpreted with caution. 

Maternal nutrition might influence the development and course of postpartum depression [17,18,41]. Previous evidence focused on the association of nutrients or individual food groups with postpartum depression with contradictory results [41,42,43,44]. Hamazaki et al. [42] found that consuming more fish and/or n-3 polyunsaturated fatty acids during pregnancy was related to a reduced risk of postpartum depression, while Nathanson et al. [41] did not observe such an association. Otherwise, supplementation with B vitamins (such as riboflavin) [43], yet not with folic acid [44], seems beneficial for postpartum depression. This inconsistency between studies could be explained by the traditional single-nutrient- or food-based approach, which may show an incomplete picture of the relationship between diet and mental health, failing to account for the interaction between nutrients and how they could contribute to depressive symptoms [41,45]. Dietary patterns, however, better reflect food and nutrient consumption and may therefore be suitable for analysis in postpartum depression epidemiology [18]. Better diet quality has been associated with a lower likelihood of depressive disorders among the non-pregnant adult population [10]. However, according to a recent review, studies examining the association between diet quality and postnatal depressive symptoms are urgently required [17].

We observed that a greater MD adherence during pregnancy was associated with lower levels of postpartum depression. Additionally, women with an optimal MD adherence (i.e., above-median) were about 72% less likely to present postpartum depression. Similarly, Chatzi et al. [46] found that women with a ‘healthy’ diet during pregnancy (14–18th g.w.) comprising vegetables, fruit, nuts, pulses, fish and seafood, olive oil, and dairy products had a 50% reduced risk of depressive symptoms at 8–10 weeks after birth. Moreover, we investigated the single components of the MD index and observed that more fruits and less red meat and subproducts was associated with less postpartum depression. Similarly, previous studies support our findings on nuts and fruits [47], as well as on red meat and subproducts [48].

Several factors, such as oxidative stress, inflammation, and changes in vascularization, might cause damage to the brain induced by diet, and these factors have been associated with the occurrence of depression [49]. A healthy diet has the potential to regulate these factors. It has been hypothesized that inflammatory processes might be behind the onset and maintenance of depressive disorders [50]. Interestingly, red meat has been associated with inflammatory biomarkers [51] and an increased risk of depression in the Spanish non-pregnant adult population [52]. On the contrary, fruit antioxidants might potentially suppress inflammation and neuronal cell damage and, subsequently, cognition [53]. Moreover, diet influences oxidative processes, which may also be implicated in the pathophysiology of depressive illnesses [54,55]. This might even compromise the brain because depression might increase the production of proinflammatory cytokines, inducing reactive oxygen species and, subsequently, triggering the lipid peroxidation process [54]. Therefore, a lower intake of pro-oxidant-rich foods (e.g., red meat and subproducts) and a greater intake of antioxidant-rich foods (e.g., fruits) could be recommended in the perinatal period [56]. In this sense, the MD could be particularly useful, since it has been showed to neutralize oxidative stress and reduce the risk of depression [57]. This could explain why an MD characterized by antioxidant nutrients, dietary fiber, and diverse fat composition (among others) could be associated with lower levels of postpartum depression.

According to previous evidence [37], exercise-only interventions during pregnancy did not clearly affect the severity of depressive symptoms at postpartum. Therefore, interventions including diet and exercise components might be more effective in preventing postpartum depression compared to exercise interventions alone. However, evidence about the interaction between diet and exercise on postpartum depression is limited. Although non-significant, participants in the intervention group with high MD adherence scored three points less in postpartum depression than the control group with low MD adherence. Our limited sample size could explain the lack of statistical significance in a relatively large effect size (70% lower risk). Therefore, our exploratory findings might suggest the potential role of diet on the exercise effects on postpartum depression, yet more research is needed about the interplay between diet and exercise in randomized controlled trials that are sufficiently powered.

This study is not free from limitations. Firstly, this is quasi-experimental study, which is a limitation, since women were not purely randomized. Notwithstanding, the randomised component was partially broken because of difficulties related to the adherence of the control group of women to their intervention regime, which represents a frequent methodological barrier in antenatal exercise research [22,23]. Secondly, postpartum depression was assessed with the self-administered EPDS, which does not relate to a clinical diagnosis of depression. Notwithstanding, the EPDS is a well-established and widely used screening tool for postpartum depression that has been validated and has high specificity and sensitivity [28]. Even though we investigated the potential moderating role of diet on the effects of exercise on postpartum depression, this study only considered diet as an observed factor; thus, the interaction effects of exercise and diet could not be determined. Further research using a 2 × 2 factorial design based on exercise and diet interventions is warranted to gain insight into this question.

Regarding strengths, participating women were followed up from the 16th g.w. until the postpartum measurement, allowing us to consider a variety of potential confounding exposures during pregnancy and early life. Additionally, we included several potential confounders, such as sociodemographic factors (age, education, and marital status), lifestyle behaviors (smoking), and other risk factors (having a diagnosis of depression or anxiety, number of abortions, gestational weight gain, and lumbar pain).

## 5. Conclusions

Overall, we found that exercise did not significantly reduce depressive symptoms at postpartum. However, a greater MD adherence, and specifically the consumption of more fruits and less red meat and subproducts, was associated with fewer depressive symptoms and less postpartum depression. The interaction between exercise and MD could lead to a lower risk of postpartum depression, although larger randomized controlled trials should formally test this hypothesis.

## Figures and Tables

**Table 1:** Sociodemographic characteristics of the study participants.

Variable	All (n = 85)	Counselling (n = 39)	Exercise (n = 46)
Age (years)	33.4 (4.2)	33.6 (4.2)	33.22 (4.2)
Mediterranean diet adherence	21.2 (4.9)	19.8 (5.2)	22.4 (4.2)
Percentage of attendance ^a^			85.5 (7.5)
Pregestational body mass index (n = 83)	23.7 (3.7)	23.0 (3.1)	24.2 (4.1)
Gestational weight gain ^b^	11.3 (4.9)	13.4 (4.5)	9.4 (4.4)
Lower back pain (VAS)	19.6 (22.9)	17.8 (23.8)	21.3 (22.3)
Postpartum depression			
Edinburgh (0–30)	5.9 (4.2)	-	-
Edinburgh ≥10 (yes, n [%])	14 (16.5)	6 (15.4)	8 (17.4)
Education	n (%)		
Low	7 (8.2)	3 (7.7)	4 (8.7)
Medium	19 (22.4)	6 (15.4)	13 (28.3)
High	59 (69.4)	30 (76.9)	29 (63.0)
Marital status			
Married/with partner	52 (61.2)	24 (61.5)	28 (60.9)
Divorced/single/widow	33 (38.8)	15 (38.5)	18 (39.1)
Working status			
Working	61 (71.8)	29 (74.4)	32 (69.6)
Not working	24 (28.2)	10 (25.6)	14 (30.4)
Number of miscarriages			
0	49 (57.6)	21(53.8)	28 (60.9)
1	26 (30.6)	10 (25.6)	16 (34.8)
2	8 (9.4)	7 (17.9)	1 (2.2)
3	2 (2.4)	1 (2.6)	1 (2.2)
Smoking (yes, n [%])	6 (7.1)	5 (12.8)	1 (2.2)
Diagnosis of depression/anxiety (yes, n [%])	2 (2.4)	1 (2.6)	1 (2.2)

Data presented as mean (SD) or frequency (%). SD, standard deviation; VAS, visual analogue scale. ^a^ When considering women on an intention-to-treat basis, the average percentage of attendance was 77.5%. ^b^ Weight at the 34th gestational week−pre-pregnancy weight.

**Table 2:** Influence of the exercise intervention on postpartum depression.

	Control Group	Exercise Group	P ^a^	P ^b^
Per-protocol basis (≥75% attendance)				
Edinburgh (n = 39 vs. 46)	6.6 (0.7)	5.3 (0.6)	0.299	0.140

Values shown as mean (standard error). ^a^ Model unadjusted. ^b^ Model adjusted for number of miscarriages.

**Table 3:** Associations of dietary habits and MD adherence during pregnancy with postpartum depression severity (n = 85).

	Longitudinal Associations	
	β	*p*
Whole-grain cereals (servings/week) †	0.023	0.830
Potatoes (servings/week) †	0.138	0.195
Fruits (servings/week) †	−0.242	0.022
Vegetables (servings/day) †	−0.058	0.599
Pulses (servings/week) †	−0.031	0.772
Fish (servings/week) †	−0.114	0.284
Red meat and subproducts (servings/week) †	0.244	0.020
Poultry (servings/week) †	−0.105	0.327
Dairy products (servings/week) †	−0.053	0.627
Olive oil (servings/week) †	−0.160	0.133
Nuts (servings/week) †	−0.054	0.616
Sweets (servings/week) †	0.026	0.806
MD adherence (4–35) †	−0.236	0.027

Each food group was introduced in a separate hierarchical regression model. Potential confounders (i.e., age, having a diagnosis of depression or anxiety, number of abortions, smoking habit, gestational weight gain, lumbar pain, marital status, and educational level) were the explanatory variables in step 1 of the stepwise regression, so that the relevant confounders were kept in the model for further analyses. Next, the explanatory variable of interest (i.e., food group or MD adherence) was entered in the model together with the covariates kept from step 1. † Adjusted for number of abortions. MD, Mediterranean diet.

## Data Availability

The datasets used and/or analyzed during the current study are available from the corresponding author on reasonable request.

## Supplementary Materials

The following supporting information can be downloaded at: https://www.mdpi.com/article/10.3390/ijerph192114450/s1, Figure S1: Flow diagram of the study participants; Table S1: Inclusion and exclusion criteria in the GESTAFIT project.
