# Supplementary material for: Exercise, Mediterranean Diet Adherence or Both during Pregnancy to Prevent Postpartum Depression—GESTAFIT Trial Secondary Analyses
Source: Int J Environ Res Public Health. 2022 Nov 4;19(21):14450. doi: 10.3390/ijerph192114450 (PMC9657805; doi:10.3390/ijerph192114450)
Supplement: Supplementary file 1 [file ijerph-19-14450-s001.zip › ijerph-1982652-supplementary.pdf]

Supplementary Material

Supplement to: **“Exercise, Mediterranean diet adherence or both during pregnancy to prevent postpartum depression. GESTAFIT trial secondary analyses”.**

Table of contents

Supplementary Figure S1. Flow diagram of the study participants.

Supplementary Table S1. Inclusion and exclusion criteria in the GESTAFIT project.

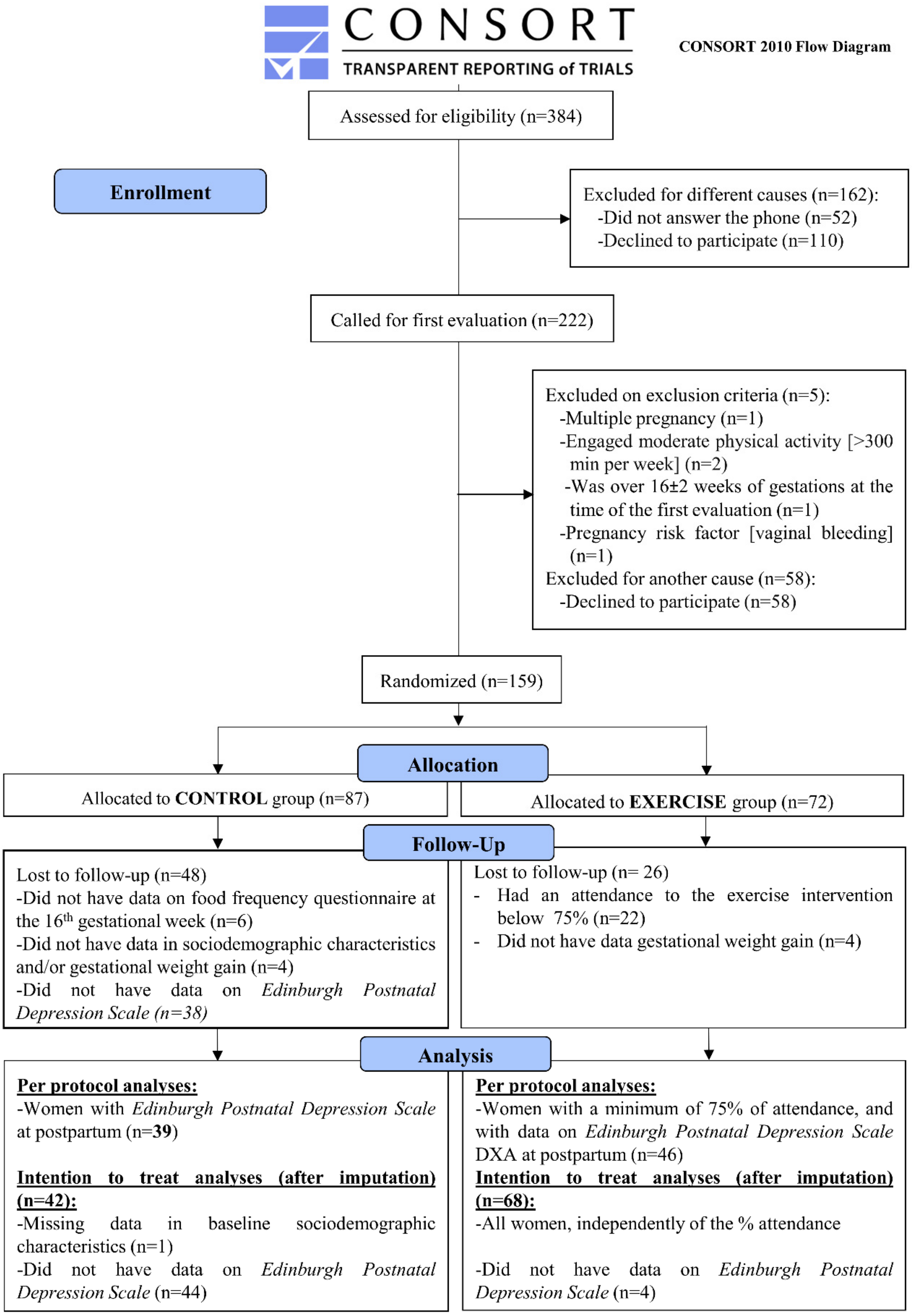

Supplementary Figure S1. Flow diagram of the study participants.

Supplementary Table S1. Inclusion and exclusion criteria in the GESTAFIT project.

**Inclusion criteria**
- Pregnant women aged 25-40 years old with a normal pregnancy course.
- Answering "no" to all questions on the PARmed-X for pregnancy.
- Being able to walk without assistance.
- Being able to read and write properly.
- Informed consent: Being capable and willing to provide written consent.
**Exclusion criteria**
- Having acute or terminal illness.
- Having malnutrition.
· Being unable to conduct tests for assessing physical fitness or exercise during pregnancy.
- Having pregnancy risk factors (such as hypertension, type 2 diabetes, etc.).
- Having a multiple pregnancy.
- Having chromosopathy or foetal malformations.
- Having uterine growth restriction.
- Having foetal death.
- Having upper or lower extremity fracture in the past 3 months.
- Suffering neuromuscular disease or presence of drugs affecting neuromuscular function.
- Being registered in another exercise program.
- Performing more than 300 minutes of at least moderate physical activity per week.
-Being engaged in another physical exercise program
- Being unwilling either to complete the study requirements or to be randomized into the control or intervention group.
